# Association between tongue pressure and oral status and activities of daily living in stroke patients admitted to a convalescent rehabilitation unit

**DOI:** 10.1002/cre2.825

**Published:** 2023-11-30

**Authors:** Shizuka Ninomiya, Wataru Fujii, Erika Matsumoto, Kiichiro Yamaguchi, Masao Hiratsuka

**Affiliations:** ^1^ Division of Dentistry Fukuoka Rehabilitation Hospital Fukuoka Japan; ^2^ Graduate School of Dentistry, Department of Oral Health Sciences Kyushu Dental University Kitakyushu Japan; ^3^ Unit of Interdisciplinary Promotion, School of Oral Health Sciences, Faculty of Dentistry Kyushu Dental University Kitakyushu Japan

**Keywords:** activities of daily living, functional independence measure, oral status, tongue pressure

## Abstract

**Background:**

Clarifying how tongue pressure in convalescent stroke patients affects oral condition and activities of daily living (ADL) is important for developing oral rehabilitation programs and for rehabilitation care to reacquire ADL.

**Objective:**

To clarify how tongue pressure is associated with oral status, ADL, and nutritional status in stroke patients.

**Methods:**

Sixty‐eight patients aged 77.9 ± 10.0 years participated. The Japanese version of the Oral Health Assessment Tool was used to evaluate oral status. Data such as the ADL index functional independence measure (FIM), nutritional intake method, serum albumin, and body mass index were extracted from medical records. To examine factors associated with tongue pressure, multiple regression analysis was performed adjusting for confounding factors. The level of statistical significance was set at *p* < .05.

**Results:**

In recovery phase stroke patients, tongue pressure was significantly lower in the total assistance group than in the partial assistance/independent group. In addition, tongue pressure was significantly lower in tube feeding patients than in oral feeding patients. FIM cognition score was an independent factor that had a significant effect on tongue pressure.

**Conclusion:**

These findings suggest that ADL status also affects tongue pressure, thus patients' ADL including the FIM cognition subscale should also be evaluated while measuring tongue pressure.

## 1 BACKGROUND

Stroke and other cerebrovascular diseases are currently the fourth leading cause of death in Japan (Vital Statistics in JAPAN ‐ Ministry of Health, Labor and Welfare, 2022), and the proportion of people aged 65 years and older who need nursing care due to stroke accounts for 15.1% of the total population (Annual Report on the Aging Society in Japan, Cabinet office, 2022). Approximately 290,000 people are estimated to experience stroke annually (Takashima et al., 2017). It is common for various disorders to affect the activities of daily living (ADL) after the disorder's onset. Therefore, it is important that patients undergo appropriate rehabilitation to live independently after a stroke.

From the standpoint of rehabilitation, stroke care is divided into acute, recovery, and maintenance phases (life stage). Although the acute phase mainly involves treating the disease, the recovery phase is focused on rehabilitation to improve motor, cognitive, and other functional disorders to prevent patients from becoming bedridden and to help them return to home life. Team‐based rehabilitation aimed at the reacquisition of ADL is particularly important during the recovery phase. This requires multidisciplinary collaboration, cooperation, and division of labor between specialists. Assigning dental hygienists to rehabilitation wards has been reported to improve rehabilitation outcomes, (Suzuki et al., 2020), and the activities of dental hygienists in rehabilitation care are expected to show even more benefits in the future. Therefore, similar to other rehabilitation staff, it is important that dental hygienists perform efficient and effective interventions, set clear goals for patients, and formulate appropriate oral rehabilitation programs grounded in evidence‐based assessments.

Among the aftereffects of stroke, dysphagia is observed at high rates (37%–78%) (Martino et al., 2005). Eating and swallowing functions involve the preparatory and oral phases in which tongue movements shape the food bolus and send it to the pharynx. Tongue movement can be evaluated as tongue pressure to provide an objective indicator of oral functions (Tsuga et al., 2011; Utanohara et al., 2008). Recently, tongue pressure measurement devices have been developed that make it easy to measure the motor functions of the tongue as a numerical value. These devices are widely used in clinical practice. Because tongue pressure can be evaluated quantitatively, it is also useful feedback to the patient. However, in stroke patients, a decline in ADL can be predicted from the influence of factors, such as hemiplegia and higher brain dysfunction, in addition to dysphagia. Because reduced ADL affects physical functions and self‐care abilities, it worsens the condition of the oral cavity (Ninomiya & Hiratsuka, 2019) and could also possibly affect tongue pressure. Furthermore, it has been reported that approximately 40% of recovery phase rehabilitation patients exhibit undernutrition at hospital admission, (Nishioka et al. 2015, 2016) hence the importance of rehabilitation based on nutritional status has been proposed. Many previous reports have shown that tongue pressure in elderly people is related to physical function and nutrition, (Chang et al., 2021; Hoyano et al., 2019; Shimizu et al., 2021) but few have examined only recovery phase stroke patients (Nakamori et al., 2021). Therefore, clarifying how tongue pressure affects oral status and ADL in recovery phase stroke patients is important for dental hygienists involved in rehabilitation care to formulate oral rehabilitation programs and may also contribute to rehabilitation care for reacquiring ADL.

Therefore, to clarify how tongue pressure is associated with oral status, ADL, and nutritional status in stroke patients admitted to a convalescent rehabilitation unit, we investigated differences in nutritional status based on the degree of ADL and oral status and examined the factors that affect tongue pressure.

## 2 METHODS

### 2.1 Study participants and materials

This study comprised 534 stroke patients admitted to a convalescent rehabilitation unit from August 1, 2018 to December 19, 2019. From them, we selected 68 patients whose attending physician had requested oral health management from the dental department, who had consciousness level I or II on the Japan Coma Scale, and who could perform the movements required to measure tongue pressure during oral assessments (43 men, 25 women, mean age 77.9 ± 10.0 [range 43–100] years). The Japanese version of the Oral Health Assessment Tool (OHAT‐J) (Chalmers et al., 2005; Matsuo & Nakagawa, 2016) was used to evaluate oral status. Data such as the ADL index functional independence measure (FIM), (Linacre et al., 1994) nutritional intake method, serum albumin (Alb [g/dL]), and body mass index (BMI [kg/m^2^]) were extracted from electronic medical records.

### 2.2 Measurement of tongue pressure

A tongue pressure measurement device (JMS Co.) was used to measure tongue pressure. After inserting the tongue pressure probe into the mouth, the subject closed the lips and gently gripped the hard ring between the upper and lower front teeth. The subject then pressed the balloon against the upper palate with the tongue continuously for 5 s. The process was explained to the patient orally and by example, and the measurements were performed after practicing several times. The measurements were performed three times and the mean was calculated. The patient's posture was dependent on the patient's condition. Taking hemodynamics into account, blood pressure, pulse rate, and percutaneous arterial oxygen saturation were measured before the measurements to ensure safety. The measurements were performed by one dental hygienist.

### 2.3 Measurement of oral status

The patient's oral status was evaluated using the OHAT‐J. There were eight assessment items: lips, tongue, gingiva/mucosa, saliva, remaining teeth, dentures, oral cleaning, and dental pain. Each was classified into three stages, from healthy to unhealthy (Matsuo & Nakagawa, 2016). Each OHAT‐J item was evaluated to calculate the total score. Two dental hygienists performed oral assessments. They confirmed the scoring in advance and fully understood it.

### 2.4 Measurement of ADL

FIM was used to evaluate the ADL; it has a 13‐item motor subscale (91 points total) that includes items on self‐care, such as eating and grooming, and on excretion and transfers. It also has a 5‐item cognitive subscale (35 points total) that includes items on social cognition. The ability to perform each item is scored from total assistance (1 point) to complete independence (7 points) for a maximum of 126 points (Linacre et al., 1994).

### 2.5 Measurement of feeding method

For the feeding method, regardless of food format, people who ate three meals a day orally were included in the oral feeding group. People on tube feeding only and those who used a combination of tube and oral feeding were included in the tube feeding group.

### 2.6 Statistical analysis

Based on the definition of the FIM motor subscale, subjects with FIM motor scores of 50 or higher were included in the partial assistance/independent group, and those with scores less than 50 were included in the total assistance group and examined using the Mann–Whitney U test. To investigate differences based on nutritional status, the subjects were assigned to the tube or oral feeding groups depending on the feeding method and were compared using the Mann–Whitney U test. To examine factors associated with tongue pressure, multiple regression analysis was performed adjusting for confounding factors. The level of statistical significance was set at *p* < .05. Statistical analysis was performed using Excel Statistics Statcel 4 (4th edition, OMS Publishinga), and the Excel add‐on software Mulcel (4th edition, OMS Publishing) was used for analysis.

### 2.7 Ethical issues

This was a retrospective study that used existing data obtained during clinical practice. The electronic medical records were viewed in the dental department office, and anonymization was performed without a correspondence table between personal information and codes/numbers to protect the confidentiality of the subjects. The study was conducted with the approval of the Fukuoka Rehabilitation Hospital Medical Ethics Committee (approval no. 2019‐D‐002) and the Kyushu Dental University Ethics Committee (approval no. 19‐80).

## 3 RESULTS

Patient attributes are shown in Table 1. There were 68 subjects, including 43 men (63.2%) and 25 women (36.8%). The types of stroke were cerebral infarction in 41 patients (60.3%), cerebral hemorrhage in 24 patients (35.3%), and subarachnoid hemorrhage in 3 patients (4.4%). The feeding methods were oral feeding in 21 patients (30.9%) and tube feeding in 47 patients (69.1%). Regarding nutritional status, the Alb value was low at 3.2 ± 0.5 g/dL, but the BMI at 21.4 ± 3.6 kg/m^2^ indicated normal body weight.

**Table 1:** Baseline characteristics of participants.

Age (years), mean ± SD		77.9 ± 10.0
Sex, *n* (%)		
	Male	43 (63.2)
	Female	25 (36.8)
Stroke type (%)		
	Brain infarction	41 (60.3)
	Brain hemorrhage	24 (35.3)
	Subarachnoid hemorrhage	3 (4.4)
FIM score, Median [IQR]		
	Motor	13 [13–28]
	Cognitive	13 [7–19]
Feeding method (%)		
	Oral feeding	21 (30.9)
	Tube feeding	47 (69.1)
Alb (g/dL), Mean ± SD		3.2 ± 0.5
BMI, (kg/m^2^), Mean ± SD		21.4 ± 3.6
mRS，Scale，Median［IQR］		4 [4, 5]

Abbreviations: Alb, albumin; BMI, Body Mass Index; FIM, functional independence measure; IQR, interquartile range; mRS, modified Rankin Scale.

Table 2 shows the distribution of the scores for each OHAT‐J item. One point was the most common score for lip, tongue, gingiva/mucosa, saliva, and oral cleaning. For caries findings in the remaining teeth, 63.2% scored 0 points, although caries findings were observed in three or fewer teeth in 26.5%. Regarding dentures, 36.7% did not wear dentures because of poor fit. Regarding oral cleaning, 94.0% scored slightly poor (1 point or higher). Almost all patients exhibited no physical or verbal signs of pain associated with dental pain.

**Table 2:** OHAT‐J distribution of each item(%).

	0	1	2
Lips	17 (25.0)	50 (73.5)	1 (1.5)
Tongue	8 (11.7)	56 (82.3)	4 (6.0)
Gums and tissues	23 (33.8)	43 (63.2)	2 (3.0)
Saliva	12 (17.6)	53 (77.9)	3 (4.5)
Natural teeth	43 (63.2)	18 (26.5)	7 (10.3)
Dentures	39 (57.3)	4 (6.0)	25 (36.7)
Oral cleanliness	4 (6.0)	48 (70.5)	16 (23.5)
Dental pain	65 (95.5)	2 (3.0)	1 (1.5)
OHAT‐J: Oral Health Assessment Tool			(*N* = 68)

**Figure 1:**
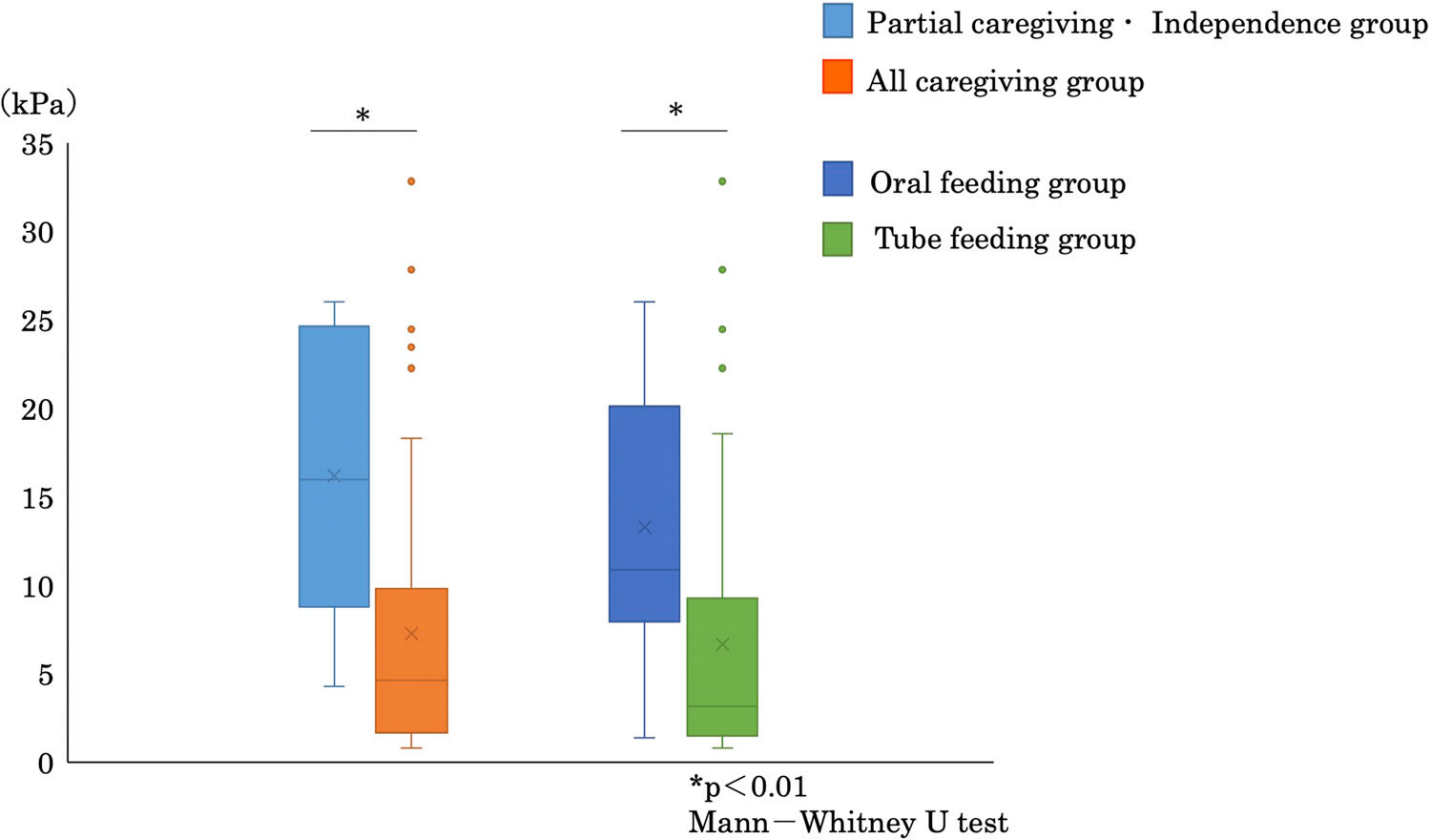
Comparison of tongue pressure based on the feeding method. Tongue pressure was significantly lower in the full assistance group than in the partial assistance/independent group (*p* < .01) (a), and in the tube feeding group than in the oral feeding group (*p* < .01) (b).

Figure 1 shows the tongue pressures in each group. The overall mean tongue pressure was 8.7 ± 8.3 kPa. In the partial assistance/independent group based on the FIM motor subscale, tongue pressure was 16.2 ± 8.0 kPa, and in the full assistance group, it was 7.3 ± 7.5 kPa. Tongue pressures were 13.3 ± 8.0 and 6.7 ± 7.6 kPa in the oral feeding and tube feeding groups, respectively. Tongue pressure was significantly lower in the full assistance group than in the partial assistance/independent group (*p* < .01) and in the tube feeding group than in the oral feeding group (*p* < .01).

Table 3 shows that multiple regression analysis was performed with all explanatory variables included to control for confounders. The results showed that the FIM cognition subscale (*β* = .330, *p* < .05) was a significant independent factor.

**Table 3:** Multiple regression analysis with tongue pressure as the objective variable.

	*B*	Standard errors	95％ CI		*β*	*p* value
Constant	−3.012	10.341	−23.697	17.673	−3.012	.772
Alb	2.647	1.852	−1.058	6.352	.158	.158
Oral feeding （yes）	3.049	2.186	−1.324	7.422	.172	.168
FIM（Motor）	0.016	0.074	−0.133	0.165	.037	.83
FIM（Cognitive）	0.317	0.123	0.072	0.562	.33	.012
OHAT‐J（Total）	−0.024	0.353	−0.73	0.681	−.007	.946
BMI	0.174	0.259	−0.343	0.692	.075	.504
mRS	−1.481	1.46	−4.401	1.44	−.163	.315

*Note*: *R* = 0.62, *R* ^2^ = 0.39, corrected degrees of freedom *R* ^2^ = 0.32, *p*＜.001.

Abbreviations: Alb, albumin; CI, confidence interval; FIM, functional independence measure; mRS, modified Rankin Scale.

## 4 DISCUSSION

In this study, we demonstrated the following regarding the three clinical questions about how tongue pressure is associated with oral status and ADL in recovery phase stroke patients. First, tongue pressure was significantly lower in recovery phase stroke patients with FIM motor scores less than 50 points (full assistance group) than in those with scores of 50 or higher (partial assistance/independent group). Second, tongue pressure was significantly lower in the tube feeding group than in the oral feeding group. Third, the FIM cognition subscale was a significant independent factor associated with tongue pressure.

In recovery phase stroke patients, tongue pressure was significantly lower in the total assistance group than in the partial assistance/independent group. We set 30 kPa as the standard for low tongue pressure, which can be caused by factors such as cerebrovascular accidents (Minakuchi et al., 2018). The overall mean tongue pressure of the subjects of the present study was 8.7 ± 8.3 kPa, which is well less than 30 kPa and is consistent with a report from the Japanese Society of Gerodontology (Minakuchi et al., 2018). The tongue is a muscular tissue, and it has been reported that a decrease in overall muscle mass may also affect tongue pressure (Maeda & Akagi, 2015). If the FIM motor subscale indicates full assistance, a decrease in overall muscle mass may also affect tongue pressure. Furthermore, in a previous study of elderly people in a nursing care facility, the tongue pressure of a walking group was significantly higher than that of a wheelchair/bedridden group, and the tongue pressure of the wheelchair group was higher than that of the bedridden group (Tanaka et al., 2015). In the present study, tongue pressure was significantly higher in the partial assistance/independent group than in the full assistance group. Our use of FIM suggests that we evaluated the ADL that the subjects were actually doing in daily life, which means the results may better reflect the lives of patients than the results of previous studies and could be useful references for setting concrete goals for patients. Our results also support those of a previous study (Shiraishi et al., 2018) that found more severe oral dysfunction with lower FIM motor scores. However, this previous study did not perform a numerical comparison of the FIM motor subscale (Shiraishi et al., 2018). The fact that FIM motor scores were significantly lower in the full assistance group than in the partial assistance/independent group suggests that this could be used as an indicator for identifying people with low tongue pressure.

Tongue pressure was significantly lower in the tube feeding group than in the oral feeding group. A previous study has also investigated the association between tongue pressure and feeding status, (Lee et al., 2016), which found similar results to our study of only recovery phase stroke patients.

Dysphagia is one of the more common aftereffects of stroke, (Martino et al., 2005) and a decrease in eating function could create a vicious cycle that leads to a decline in tongue function. In patients who do not eat orally, the oral cavity tends to become immobile, which also affects tongue pressure. Therefore, tongue pressure strength training at an early stage is necessary. Because strengthening tongue pressure can improve and prevent the decline in oral and swallowing phase functions, (Lee et al., 2016) it is even more important to evaluate tongue pressure in tube feeding patients, which should be performed early in the process of devising an oral rehabilitation program.

In the present study, multiple regression analysis was performed to extract the factors that affect tongue pressure. The results showed that the FIM cognition subscale was an independent factor significantly associated with tongue pressure. Previous studies have demonstrated that cognitive decline affects a variety of physical functions, including ADL (Kamo et al., 2019). Furthermore, Kamo et al. have reported that when the FIM cognition score is low, the amount of improvement that can be expected in the FIM motor subscale is extremely small (Kamo et al., 2019). Tongue pressure can be easily and quantitatively measured using a device, but patients with cognitive decline can have difficulty performing the voluntary task of lightly gripping the hard ring of the probe with the upper and lower front teeth and then continuously pressing the balloon against the palate with the tongue for 5 s. It should be noted that patients with low FIM cognition scores may have lower tongue pressure measurements. In addition, ADL assessments such as the FIM cognition subscale should be considered when measuring tongue pressure, but because such assessments can be difficult for dental hygienists who are not constantly observing patients' daily lives, a multidisciplinary approach is extremely important.

In addition, our study showed that oral status had no effect on tongue pressure. It has been reported that recovery phase stroke patients are more likely to have poor oral status if they have reduced ADL (Ninomiya & Hiratsuka, 2019). However, we could not find any studies reporting an association between poor oral status and tongue pressure. Going forward, we plan to increase the sample size to conduct further studies.

One limitation of this study is that the posture during the tongue pressure measurements was adjusted for each individual patient, thus the measurement conditions were not standard. Hori et al. have reported objective findings during tongue movements, showing that tongue pressure is greatly affected by posture (Hori et al., 2011). However, we did not examine the effects of posture. In the future, further studies should be conducted under standardized conditions.

## 5 CONCLUSION

In stroke patients admitted to a convalescent rehabilitation unit, tongue pressure was significantly lower in the total assistance group (FIM motor score <50) than in the partial assistance/independent group (FIM motor score ≥50 points). In addition, tongue pressure was significantly lower in tube feeding patients than in oral feeding patients. Finally, the FIM cognition score was an independent factor that had a significant effect on tongue pressure. These findings suggest that ADL status also affects tongue pressure, thus patients' ADL including the FIM cognition subscale should also be evaluated while measuring tongue pressure.

## AUTHOR CONTRIBUTIONS

Shizuka Ninomiya was involved in the conception of the study, acquisition of data, data entry, interpretation of results, and drafting of the manuscript. Erika Matsumoto was involved in the conception of the study, acquisition of data, interpretation of results, and drafting of the manuscript. Kiichiro Yamaguchi was involved in patient screening and the acquisition of data. Masao Hiratsuka was involved in the conception of the study and interpretation of results. Wataru Fujii served as senior author and was involved in the conception of the study, interpretation of results, and drafting of the final version of the manuscript.

## CONFLICT OF INTEREST STATEMENT

The authors declare no conflicts of interest.

## Data Availability

Data are available on request from the authors.
